# Front-Line Window Therapy with Temozolomide and Irinotecan in Patients with Primary Disseminated Multifocal Ewing Sarcoma: Results of the ISG/AIEOP EW-2 Study

**DOI:** 10.3390/cancers13123046

**Published:** 2021-06-18

**Authors:** Sebastian Dorin Asaftei, Nadia Puma, Anna Paioli, Marco Petraz, Carlo Morosi, Marta Podda, Angela Tamburini, Emanuela Palmerini, Luca Coccoli, Giovanni Grignani, Carla Manzitti, Rossella Bertulli, Francesco De Leonardis, Marco Rabusin, Anna Campello, Elisa Tirtei, Piero Picci, Arcangelo Prete, Alessandra Longhi, Franca Fagioli, Roberto Luksch

**Affiliations:** 1 Pediatric Onco-Hematology, A.O.U Città della Salute e della Scienza, University of Turin, Piazza Polonia, 94, 10126 Turin, Italy; anna.campello@unito.it (A.C.); elisa.tirtei@unito.it (E.T.); franca.fagioli@unito.it (F.F.); 2 Pediatric Oncology Unit, Fondazione IRCCS Istituto Nazionale dei Tumori, Via Giacomo Venezian, 1, 20133 Milan, Italy; Nadia.Puma@istitutotumori.mi.it (N.P.); marta.podda@istitutotumori.mi.it (M.P.); roberto.luksch@istitutotumori.mi.it (R.L.); 3 Chemotherapy Unit, IRCCS Istituto Ortopedico Rizzoli, Via Giulio Cesare Pupilli, 1, 40136 Bologna, Italy; anna.paioli@ior.it (A.P.); emanuela.palmerini@ior.it (E.P.); alessandra.longhi@ior.it (A.L.); 4 Pediatric Radiology, A.O.U Città della Salute e della Scienza, Piazza Polonia, 94, 10126 Turin, Italy; mpetraz@cittadellasalute.to.it; 5 Radiology Department, Fondazione IRCCS Istituto Nazionale dei Tumori, Via Giacomo Venezian, 1, 20133 Milan, Italy; carlo.morosi@istitutotumori.mi.it; 6 Pediatric Onco-Hematology Unit, Centro di Eccellenza di Oncologia ed Ematologia, AUOM, Viale Gaetano Pieraccini, 24, 50139 Florence, Italy; angela.tamburini@meyer.it; 7 Pediatric Hematology Oncology Unit, S. Chiara-Pisa University Hospital AOUP, Via Bonanno Pisano, 10, 56126 Pisa, Italy; l.coccoli@ao-pisa.toscana.it; 8 Department of Medical Oncology, Candiolo Cancer Institute FPO-IRCCS, SP 142, km 3,95, 10060 Candiolo, Italy; giovanni.grignani@ircc.it; 9 Department of Haematology-Oncology, IRCCS G. Gaslini Children’s Hospital, Via Gerolamo Gaslini, 3, 16147 Genoa, Italy; carlamanzitti@ospedale-gaslini.ge.it; 10 Adult Mesenchymal Tumor Medical Oncology Unit, Fondazione IRCCS Istituto Nazionale dei Tumori, Via Giacomo Venezian, 1, 20133 Milan, Italy; rossella.bertulli@istitutotumori.mi.it; 11 Division of Pediatric Haematology Oncology, University Hospital, Piazza Giulio Cesare, 11, 70124 Bari, Italy; francesco.deleonardis@policlinico.ba.it; 12 Department of Pediatrics, Institute for Maternal and Child Health, IRCCS Burlo Garofolo, Via dell’Istria, 65, 34137 Trieste, Italy; marco.rabusin@burlo.trieste.it; 13 Italian Sarcoma Group, Via Cà Ricchi, 33, 40068 San Lazzaro di Savena, Italy; piero.picci@italiansarcomagroup.org; 14 Pediatric Hematology and Oncology Unit, S.Orsola-Malpighi Hospital, Via Giuseppe Massarenti, 9, 40138 Bologna, Italy; arcangelo.prete@aosp.bo.it

**Keywords:** primary disseminated multifocal Ewing sarcoma, temozolomide, irinotecan, front-line treatment

## Abstract

**Simple Summary:**

The prognosis of patients with primary disseminated multifocal Ewing sarcoma (PDMES) remains dismal. Previously, a combination of temozolomide and irinotecan (TEMIRI) was tested in patients with refractory or relapsed disease. The aim of our study was to evaluate the activity and tolerability of TEMIRI (two courses) as a front-line treatment in PDMES. With thirty-four patients enrolled, the Response Evaluation Criteria in Solid Tumors (RECIST), after TEMIRI, was acceptable with a manageable toxicity and a high percentage of patients showing an amelioration in their Eastern Cooperative Oncology Group (ECOG)/Lansky scores. TEMIRI in front-line therapy showed encouraging activity and deserves further evaluation combined with conventional treatments in non-metastatic patients; meanwhile, new treatment strategies for PDMES are urgently needed.

**Abstract:**

Purpose: The main objective was to evaluate the activity and tolerability of TEMIRI as a front-line treatment in primary disseminated Ewing sarcoma (PDMES) using the RECIST 1.1 criteria. The secondary objectives included the assessment of toxicity and the performance status/symptom changes. Methods: Between 2012 and 2018, patients with PDMES received two courses of temozolomide 100 mg/sqm/day + irinotecan 50 mg/sqm/day for 5 days every 3 weeks as an amendment to the Italian Sarcoma Group/Associazione Italiana EmatoIogia ed Oncologia Pediatrica (ISG/AIEOP) EW-2 protocol (EUDRACT#2009-012353-37, Vers. 1.02). Results: Thirty-four patients were enrolled. The median age at diagnosis was 19 years (range 3–55). After TEMIRI, the RECIST response was as follows: a partial response in 20 (59%) patients, stable disease in 11 (32%), and disease progression in 3 (9%). The ECOG/Lansky score was improved in 25/34 (73.5%) cases, and a reduction or disappearance of pain was observed in 31/34 patients (91%). The incidence of grade 3–4 toxicity was 3%. The 3-year event-free survival (EFS) and overall survival (OS) were 21% (95% CI 6–35%) and 36% (95% CI: 18–54%), respectively. Conclusion: the smooth handling and encouraging activity demonstrated by up-front TEMIRI did not change the EFS in PDMES, so this result suggests the need for the further evaluation of the efficacy of TEMIRI in combination with conventional treatments in non-metastatic patients.

## 1. Introduction

Ewing sarcoma (EWS) is a high-grade sarcoma arising in bone or soft tissue with a peak of incidence in adolescents and young adults. The presence of metastases at diagnosis occurs in about 25% of patients and is the most relevant negative prognostic factor [1,2,3].

The prognosis of patients with metastases limited to the lungs, despite the use of several intensive therapeutic approaches, remains poor, with the probability of 5-year event-free survival (EFS) being around 45% [4,5]. In patients treated according to the ISG/SSG IV protocol, the 5-year EFS was 43% and the overall survival (OS) rate was 52%, with an intensive approach including myeloablative chemotherapy and total lung irradiation [4]. The prognosis of patients with multiple skeletal metastases and/or bone marrow infiltration, with/without lung/pleural metastases, defined as primary disseminated multifocal Ewing sarcoma (PDMES), remains even poorer. In the Euro-Ewing 99 Trial, the 3-year EFS and survival estimates for PDMES were 27% ± 3% and 34% ± 4%, respectively [6].

Irinotecan, a camptothecin prodrug form of SN-38, is an inhibitor of topoisomerase I, an enzyme responsible for variation in the form of DNA during replication and transcription. The inactivation of this enzyme by irinotecan results in single-strand breaks in DNA and causes S-phase-specific cytotoxicity. Temozolomide is an alkylating agent which promotes cytotoxicity primarily via the O6-methylation of guanine, leading to a base-pair mismatch and the possible inhibition of DNA replication.

Combination treatment with temozolomide plus irinotecan (TEMIRI) has shown antineoplastic activity in different solid tumors, including brain tumors, neuroblastoma, and EWS [7,8,9,10]. In preclinical studies, it has been demonstrated that the activity of the combination of irinotecan and temozolomide was significantly greater than the activity of either agent administered alone. Although it is not clear how the cytotoxicity of irinotecan is potentiated by temozolomide, it may be correlated with the effect of temozolomide-induced DNA methylation, which could lead to the localization and enhancement of topoisomerase I cleavage complexes, allowing irinotecan to more effectively stabilize the DNA–enzyme complexes [11,12]. This therapeutic synergy is greater when temozolomide is given 1 h before the administration of irinotecan [13]. In addition to this synergistic activity, the advantage of the combination lies in their non-overlapping toxicity profiles (diarrhea vs. myelosuppression) and different resistance mechanisms.

TEMIRI has been used in adult and pediatric patients with recurrent EWS with an overall response rate (ORR) ranging between 28% and 63% [14,15,16,17,18,19,20]. ECOG and LDH were found to be predictors of response to TEMIRI and were factors independently associated with progression-free survival (PFS) and overall survival (OS) [19]. The role of irinotecan in EWS has also been investigated in a phase II window study in a chemo-naïve population of patients with extra-pulmonary metastatic disease, suggesting a modest degree of activity for this drug in a front-line therapy setting as well (ORR 24%), thus highlighting the potential role of TEMIRI in the management of EWS [21]. The pragmatic choice to use a 5-day 50 mg/sqm/day irinotecan dose was made given the reported activity, toxicity, and shorter hospitalization time data [17].

Thus, the activity and toxicity profile of TEMIRI reported in the literature suggests that this combination might be added to conventional chemotherapy combinations in the first-line therapy setting in PDMES in order to increase the survival rate.

The aim of this study is to assess the safety and efficacy of TEMIRI therapy in patients with PDMES at diagnosis as a front-line treatment.

## 2. Patients and Methods

In 2012, the Italian Sarcoma Group (ISG) and the Associazione Italiana Ematologia Oncologia Pediatrica (AIEOP) began a study for patients with PDMES at onset (EUDRACT#2009-012353-37, Vers. 1.02). The ISG/AIEOP EW-2 protocol study was tailored for the treatment of patients with EWS and lung metastases, consisting of the ISG/SSG IV protocol with the addition of a maintenance phase with oral cyclophosphamide and celecoxib. The protocol was then amended to add 2 courses of TEMIRI for frontline patients with PDMES. All patients underwent a biopsy to provide histological confirmation and the molecular analysis of each specimen. The initial evaluation included Computed Tomography (CT) and/or Magnetic Resonance Imaging (MRI) of the site of the primary tumor, thoracic CT, total-body 99-TC scan and/or PET-TC, bone marrow aspirates, complete blood chemistry (also including the serum lactate dehydrogenase (LDH) level), echocardiography, electrocardiogram (ECG), and the assessment of pain according to the Numeric Pain Rating scale. The histological diagnosis and radiological evaluation, both before and after the front-line therapy, were centrally reviewed. All ethical committees of the participating institutions approved the protocol, and written informed consent was obtained from all patients or their legal guardians.

The up-front therapy consisted of 2 courses of oral temozolomide (100 mg/sqm/day) and intravenous irinotecan (50 mg/sqm/day) for 5 consecutive days (days 1–5) every 21 days. Temozolomide was administered orally 1 h before irinotecan. The second course of TEMIRI was started if the absolute neutrophil count (ANC) was >1000/µL, the platelet count was >100,000/µL, and no hepatic toxicity (serum transaminases >5n of normal value) was noticed. Dose adjustments were made if hematological toxicities or diarrhea persisted after day 21 (temozolomide 75 mg/sqm and irinotecan 40 mg/sqm, respectively). To prevent irinotecan-associated diarrhea, 8 mg/kg/day of prophylactic cefixime was administered 2 days prior to irinotecan therapy and continued until the completion of the cycle; delayed diarrhea was managed using loperamide when necessary.

After completing 2 courses of TEMIRI, all patients underwent a restaging of the disease by performing an MRI and/or a CT scan of the primary site, thoracic CT, 99-TC bone scan or PET-TC, and complete blood chemistry. The subsequent treatment program started 21 days after completing the up-front therapy and consisted of an induction phase and surgery and/or radiotherapy (RT) at the site of the primary tumor, followed by a consolidation phase with myeloablative therapy in patients with partial/complete remission and a subsequent maintenance phase with oral cyclophosphamide and celecoxib (Table 1).

The main objective was to test the activity of TEMIRI according to the Response Evaluation Criteria in Solid Tumors (RECIST) 1.1 [22]. All target lesions had a soft tissue component that was accurately measured in the central review. The overall response rate (ORR), defined as the percentage of evaluable patients with complete response (CR) or partial response (PR), was the main indicator of effectiveness. Secondary objectives included an assessment of the toxicity profile and the clinical benefit (measured using performance status and pain scale tools) of the combination. The performance status was evaluated using the Lansky score for patients less than 12 years old and the Eastern Cooperative Oncology Group (ECOG) scale for patients aged 12 years or older. Pain measurement was performed with a Numeric Pain Rating Scale (NPRS) for patients over 10 years of age and a Wong–Baker FACES Pain Rating Scale for patients less than 10 years old. Toxicity was registered after each cycle according to the Common Toxicity Criteria for Adverse Events (CTCAE) version 3.0.

A two-step study design by Simon was planned, with the consecutive enrollment of 12 patients for the first stage (group 1). If a major response was observed in at least 4 patients from group 1, 18 more patients were consecutively enrolled; otherwise, the combination TEMIRI had to be considered ineffective and the enrollment interrupted. The provided sample size of 30 patients guaranteed a statistical power of 80% through a one-tailed test at a significance level of 10%, assuming that an ORR ≤40% is unacceptable, while an ORR ≥70% is indicative of highly efficient treatment. The choices of a one-tailed test and a significance level of 10%, higher than the conventional value of 5%, were justified by the fact that this was a pilot study and PDMES is a very rare condition.

All patients were followed up to assess their overall survival (OS), taken as the time from starting TEMIRI to death or at the latest follow-up, and EFS, defined as the time from starting TEMIRI to the first occurrence of tumor progression/recurrence after response or death from any cause. The Kaplan and Meier method was used to estimate survival curves.

## 3. Results

Between May 2012 and May 2018, 34 consecutive patients with PDMES were enrolled in the study. Patient characteristics are shown in Table 2. The median age at diagnosis was 19 years (range 3–55); the male/female ratio was 2.4. Most of the patients presented with multifocal bone and lung metastases at diagnosis (53%), 35% with multifocal/unifocal bone metastases, while only a minority also presented with pleural metastases (12%). Bone marrow involvement was evaluated in 10/34 patients. From the molecular point of view, the patients were divided as follows:_29 patients with EWS-FLI1 t(11;22)(q24;q12), 4 patients with EWS-ERG t(11;22)(q22;q12), and 1 patient with EWS-ETV1 t(7;22)(q22;q12). This distribution confirms the literature data and cannot be correlated with the survival data. At diagnosis, 65% of the patients had moderate to severe pain at the site of the primary tumor and/or at sites of metastases, resulting in restrictions to daily activities and selfcare (ECOG score ≥2 in 70% of the series). Pain lasted for more than 3 months in 53% and the LDH level was elevated in about half of the patients.

All patients received the two up-front cycles of TEMIRI as scheduled, with a total of 68 cycles administered, and all were eligible for response evaluation. Table 3 shows the clinical, hematological, and radiological responses of the 34 patients. An ORR of 59% was achieved, with PR observed in 20 patients, stable disease (SD) in 11 patients (32%), and progression of the disease (PD) in 3 patients (9%). The best responses were recorded in two patients who experienced the complete disappearance of the soft tissue component of the primary tumor, while the other two patients experienced a complete remission of their lung metastases. After TEMIRI, the amelioration of the ECOG score was achieved in 25 patients (73.5%), and the reduction in or disappearance of pain was observed in 31 patients (91%). A normalization of the LDH levels was recorded in 12 patients with PR, 6 patients with SD, and 1 patient with PD, and in 90% of them the pathological LDH value fell to within regular levels.

TEMIRI toxicity was evaluable in 67/68 (= 98.5%) of the course. The incidences of grade 3–4 non-hematological and hematological toxicity were noted in 3% and 3%, respectively: one patient experienced grade 4 neutropenia and thrombocytopenia and one patient experienced grade 3 diarrhea. The dose adjustment of TEMIRI was applied in three patients with grade 1–2 diarrhea, and their serum bilirubin was increased. Admission to hospital, mainly due to diarrhea grade 1–2, was necessary in 10 patients (14/68 courses), and most of them were children and adolescents aged < 18 y. Patients with grade 1–2 diarrhea were admitted to receive supportive care in a day hospital setting in line with the local policy of the institutions.

**Figure 1:**
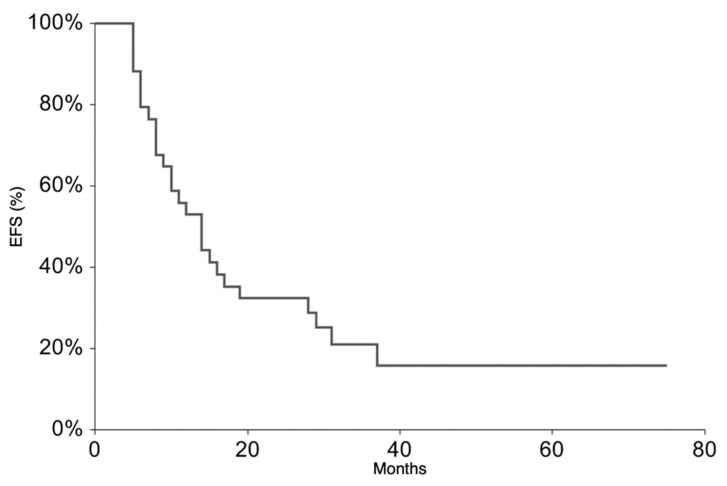
Event-Free Survival for the whole patient group.

**Figure 2:**
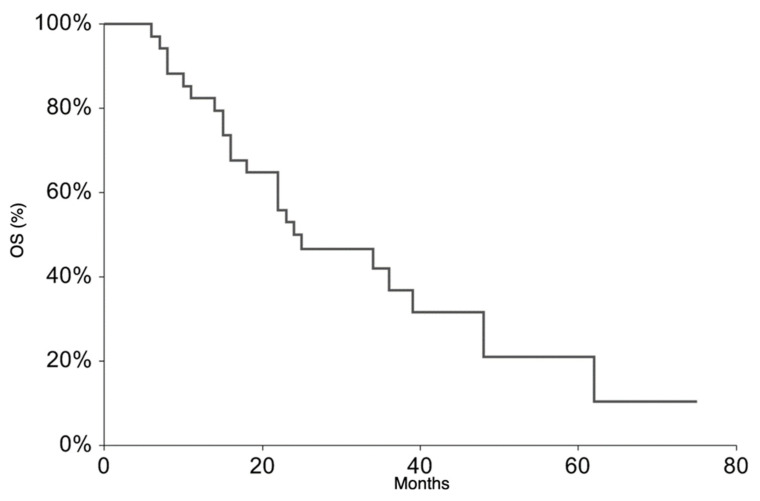
Overall Survival for the whole patient group.

All patients continued the treatment according to the ISG/AIEOP EW-2 study protocol. The EFS rate at 3 years was 20.9% (95% CI 6.3–35.6) (Figure 1). The 3-year EFS probability appeared to be significantly higher in patients who experienced PR + SD (23%, 95% CI 7.1–38.8%) than in those who did not (0%, *p*-value = 0.007). The OS rate at 3 years was 36.5% (95% CI 18.4–54.6%) *(*Figure 2). At the time of the present analysis, 11 patients are alive and 7 are in complete remission.

In the univariate analysis, there were no significant differences in 3-year EFS and 3-year survival probabilities according to age, sex, site of primary tumor, pattern of metastases, or serum LDH at diagnosis (Table 4). After multivariate analysis, the tumor response after TEMIRI (hazard ratio (HR) = 0.05; *p*-value = 0.003), normal serum LDH level (HR = 0.02; *p*-value = 0.01), and a high disease load at diagnosis (bone + lung + pleural metastases at diagnosis) (HR = 9.2; *p*-value = 0.02) were noted to be factors independently associated with survival.

## 4. Discussion

This multicentric study investigated the role of the combination of temozolomide and irinotecan, as an up-front window therapy, in a prospective series of patients with PDMES.

Among the studies involving PDMES, the most encouraging data came from the Euro-EWING 99 trial, which outlined 3-year EFS and OS rates of 31% and 37%, respectively, for patients with bone + lung metastases at diagnosis [6], and from the study by the Société Francaise des Cancers de l’Enfant that described a 5-year OS of 36% for patients with bone metastases only [23]. In both study protocols, treatment consisted of seven cycles of induction chemotherapy, local therapy at the site of the primary tumor, and high-dose chemotherapy (BuMel) with autologous stem-cell rescue treatment.

In order to improve the survival probability in PDMES, different studies have experimented with different pharmacological combinations as up-front window therapy. In 2007, ISG published the results of an up-front window therapy with melphalan in patients with previously untreated PDMES [24]. Despite the high response rate to melphalan (ORR 79%), the study concluded that the use of up-front melphalan had no favorable impact on outcome, and death within 3 years was observed in almost all patients. Moreover, this therapy was burdened with grade 3–4 cytopenia in the majority of patients. Bernstein et al. [25] assessed the efficacy of topotecan with/without cyclophosphamide as window therapy, demonstrating that topotecan was much more active in combination (ORR 56.8%) than alone (ORR 8.3%). Nevertheless, the EFS and OS rates were not significantly different for patients who received topotecan or did not. In a phase II Euro-EWING study, irinotecan was administered as window therapy [21]. In this study, an ORR of 24% was reported, with 29% of patients showing disease progression, concluding that irinotecan as a single agent was not recommended for patients with EWS. Finally, an ORR of 8% and no impact on survival were obtained by administering two cycles of cisplatin, as front-line window therapy, in an ISG study in patients with PDMES [26].

TEMIRI has been widely studied over the past 10 years in the setting of relapsed/recurring EWS in non-randomized studies, with a cumulative response rate of 47% reported in seven studies for a total of 166 patients (range 28–65%) [14,15,16,17,18,19,20,27]. The enthusiasm for the activity of TEMIRI in resistant/relapsed Ewing sarcoma was dampened by the results of the ongoing Euro-EWING Consortium study rEECur (EUDRACT#2014-000259-99), where the primary outcome was the objective response to different drug combinations that were evaluated in a randomized multi-arm-multi-stage fashion. In fact, the overall response rate in the TEMIRI arm was 20%, which was slightly worse than in the other arms (topotecan + cyclophosphamide or high-dose ifosfamide), where the overall response rate was 23%. For this reason, according to the protocol design the TEMIRI arm was terminated. In comparison with the other combinations, TEMIRI caused higher gastrointestinal toxicity, but the side effects were counterbalanced by a clearly lower rate of myelosuppression, febrile neutropenia, and infections [28].

It should be noted that there is another ongoing study using TEMIRI associated with standard chemotherapy (five drugs regimen) in newly diagnosed EWS (NCT01864109).

To date, as far as we know this is the first study reporting the role of TEMIRI in newly diagnosed EWS, and, not surprisingly, the results in activity are different from those reported in the relapsed setting in the rEECur study. Upfront TEMIRI courses showed encouraging activity, with 59% ORR and PD observed in only 9% of patients. A clinical benefit of TEMIRI was documented, with an improvement in the ECOG score achieved in 73.5% of patients and the reduction in or disappearance of pain in 91% of patients. The toxicity was manageable, with only one patient reporting grade 3–4 non-hematological and hematological toxicity, respectively. Even if 10/34 patients experienced diarrhea, this side effect never exceeds grade 2, and only in four cases was diarrhea managed through hospitalization. Only three patients with grade 1–2 diarrhea required dose adjustment of TEMIRI.

This low hematological toxicity renders TEMIRI unique in comparison to the conventional chemotherapies available for the treatment of Ewing sarcoma.

Despite the high response rate to TEMIRI, its addition to the intense backbone therapy included in the ISG/AIEOP EW-2 protocol did not impact on survival. The 2-year EFS and OS rates were 32.3% and 50%, respectively, decreasing to 21% and 36.7%, respectively, 3 years after diagnosis. The rates are therefore not significantly different from those previously reported [6,23]. The assessment of bone marrow involvement in all patients was not feasible. This could represent a bias in this study and could partially undervalue the disease burden of the case series leading to the underestimation of the EFS and OS rates; in fact, patients with both bone and bone marrow involvement have been shown to have a very poor prognosis [2,6,23].

## 5. Conclusions

Two courses of front line TEMIRI showed encouraging activity with a 59% ORR, but they had no impact on the outcome. New treatment strategies are urgently needed in PDMES, and attempting to intensify international cooperation to investigate new drugs in such a rare condition is the right way forward. We are aware that the negative results obtained in the relapsed setting in the rEECur Study will dampen the probability of the inclusion of TEMIRI in future prospective trials. Nevertheless, considering its favorable toxicity profile in first-line therapy, as demonstrated in the present study, TEMIRI could be considered a good alternative in the treatment of Ewing sarcoma in patients with fragile hemopoiesis that hampers the use of conventional myelotoxic regimens.

## Figures and Tables

**Table 1:** Treatment program for patients with PDMES (ISG/AIEOP EW-2 Vers. 1.02).

ISG/AIEOP EW-2, Vers 1.02 Protocol													
0–3 w	6 w	9 w	12 w	15 w		18 w	21 w	24 w	27 w	30 w			to 60 w
TEMIRI × 2	VAI	CE	VAI	CE	Surgery	VAC	IE	VAC	IE	BU-MEL	PBSCT	TLI	CEL-CYC
						Radiotherapy							

Front-line window therapy: 2 cycles of Temozolomide (100 mg/sqm/day + Irinotecan (50 mg/sqm/day). VAI = Vincristine (1.4 mg/sqm) + Adriamycin (90 mg/sqm) + Ifosfamide (9 gr/sqm) CE = Cyclophosphamide (4 g/sqm) + Etoposide (600 mg/sqm) Radiotherapy (42–54 Gy) IE = Ifosfamide (9 gr/sqm) + Etoposide (300 mg/sqm); VAC = Vincristine (1.4 mg/sqm) + Adriamycin (80 mg/sqm) + Cyclophosphamide (1.2 g/sqm); Bu-MEL = Busulfan (0.8–1.2 mg/Kg i.v.) + Melphalan (140 mg/sqm) + autologous stem cell rescue. TLI = total lung irradiation (only for patients with lung metastasis). CEL-CYC = Maintenance therapy with 6 months oral Celecoxib 400 mg bid (250 mg/sqm bid for pts < 14 years old) + Cyclophosphamide 50 mg/sqm (35 mg/sqm/day if <14 years old).

**Table 2:** Patient Characteristics.

Characteristics	*n* (%)
Sex	
Male	24 (70)
Female	10 (30)
Age	
≤14 y	11 (32)
15–18 y	8 (23)
19–24 y	10 (30)
≥25	5 (15)
Primary tumor location	
Pelvis	16 (47)
Extremity	10 (30)
Axial	3 (8)
Other	5 (15)
Pattern of metastases at initial diagnosis	
Bone	12 (35)
Bone + Lungs	18 (53)
Bone + Lungs + Pleural	4 (12)
Lung metastases	
≤3 nodules	6 (27.5)
>3 nodules	16 (72.5)
ECOG score	
0	5 (15)
1	5 (15)
2	14 (41)
3	7 (20)
4	3 (9)
LDH	
Normal	16 (47)
High	18 (53)
Pain (Numeric Pain Rating scale)	
≤5	7 (20)
>5	22 (65)
n.a.	5 (15)

**Table 3:** Clinical, hematological and radiological responses to treatment. PR = Partial Response, SD = Stable Disease, PD = Progression Disease.

Patient ID#	Age	Primary Site	Bone Metastasis	Lung Metastasis	Pleural Metastasis	Tumor Response	ECOG Pre-TEMIRI	ECOG After 2 Courses TEMIRI	Pain Before TEMIRI	Pain After 2 Courses TEMIRI
1	12	Scapula	Multifocal	Yes	Yes	SD	4	4	9	9
2	21	Scapula	Multifocal	Yes	No	PD	0	0	5	0
3	22	Pelvis	Multifocal	No	No	PD	4	4	8	8
4	17	Femur	Multifocal	Yes	No	PR	2	0	7	0
5	36	Femur	Multifocal	No	No	PR	3	1	9	2
6	20	Pelvis	Multifocal	Yes	No	SD	3	1	9	3
7	27	Femur	Multifocal	No	No	PR	1	0	5	0
8	21	Vertebra	Multifocal	Yes	No	PR	0	0	5	0
9	14	Foot	Multifocal	No	No	SD	2	1	7	0
10	18	Femur	Multifocal	Yes	Yes	PR	2	1	9	0
11	12	Pelvis	Multifocal	Yes	No	PR	2	1	8	2
12	23	Pelvis	Multifocal	Yes	No	PD	3	2	9	4
13	12	Vertebra	Multifocal	No	No	SD	3	1	6	2
14	27	Pelvis	Multifocal	No	No	PR	2	0	8	0
15	10	Pelvis	Multifocal	No	No	PR	2	0	9	0
16	16	Pelvis	Multifocal	No	No	PR	3	1	9	2
17	20	Pelvis	Multifocal	Yes	No	PR	3	0	10	0
18	16	Pelvis	Unifocal	Yes	No	PR	2	1	8	4
19	29	Fibula	Multifocal	Yes	No	PR	2	0	8	0
20	24	Pelvis	Multifocal	Yes	No	SD	0	0	5	0
21	13	Pelvis	Multifocal	Yes	No	SD	0	0	0	0
22	18	Pelvis	Multifocal	Yes	No	PR	1	1	0	0
23	22	Scapula	Multifocal	Yes	Yes	SD	2	1	4	2
24	20	Pelvis	Multifocal	Yes	No	SD	0	0	3	0
25	55	Vertebra	Multifocal	No	No	PR	1	1	5	2
26	16	Fibula	Multifocal	Yes	No	SD	2	0	7	0
27	12	Humerus	Multifocal	No	No	PR	2	1	5	0
28	14	Pelvis	Multifocal	No	No	SD	1	0	3	0
29	15	Pelvis	Multifocal	Yes	No	PR	2	0	7	2
30	16	Pelvis	Multifocal	Yes	Yes	PR	3	1	9	2
31	21	Tibia	Multifocal	Yes	No	SD	4	1	10	2
32	10	Clavicle	Multifocal	No	No	PR	2	0	6	0
33	3	Femur	Multifocal	Yes	No	PR	2	0	8	2
34	10	Rib	Multifocal	Yes	No	PR	1	0	8	3

**Table 4:** Univariate analysis for Overall Survival (OS) and Event Free Survival (EFS).

Variable	*n*	3 Year OS				3 Year EFS			
		Events	Cumulative OS (%)	95% CI	*p*-Value	Events	Cumulative EFS (%)	95% CI	*p*-Value
Sex									
Female	10	6	50	19–80.9	0.7	9	13.3	0–36.9	0.7
Male	24	21	28.2	5–50.9		21	15.5	0–32.7	
Age									
<18y	19	14	41.3	16.3–66.4	0.5	16	22.1	1.6–42.6	0.6
>18y	15	13	31.1	6.7–55.5		14	20	0-40.4	
Recist Response									
PR + SD	31	24	40	20.6–59.4	0.007	27	23	7.1–38.8	0.05
PD	3	3	0	0		0	0	0	
Primary Tumor Site									
Pelvis	16	14	21.8	0–54.5	0.8	16	0	0	0.9
Extremity	10	8	28	0–60.1		8	30	1.6–58.4	
Axial	3	2	33.3	0–86.7		3	0	0	
Other	5	3	40	0–82.9		4	20	0–55	
Metastatic Pattern									
Bone	12	7	41.7	13.7–69.5	0.4	10	16.7	0–37.7	0.9
Bone + Lung	18	17	25	0–61.5		17	22.2	0–45.2	
Bone + Lung + Pleural	4	3	25	0–67.4		3	25	0–67.4	
LDH Level at Diagnosis									
Normal	16	13	46.4	14.5–78.3	0.1	13	23.3	0–48.2	0.1
High	18	14	25.9	4.9–46.9		15	16.7	0–33.9	

## Data Availability

Data collection was performed through an eCRF by the ISG: www.isg-area-riservata.org/dh/ (Last Accessed on 14 May 2021).
